# Sex-specific functional evolution of *Dmrt1* in African clawed frogs (*Xenopus*), and the importance of genetic tipping points in developmental biology

**DOI:** 10.1371/journal.pgen.1011992

**Published:** 2026-01-02

**Authors:** Lindsey M. Kukoly, Sarah R. Porter, Danielle C. Jordan, Heather A. Murphy, Martin Knytl, Nikko Shaidani, William R. Thomas, Carl Anderson, Ian Dworkin, Marko E. Horb, Ben J. Evans

**Affiliations:** 1 Biology Department, Life Sciences Building, McMaster University, Hamilton, Canada; 2 Eugene Bell Center for Regenerative Biology and Tissue Engineering and National Xenopus Resource, Marine Biological Laboratory, Woods Hole, Massachusetts, United States of America; 3 The School of Biological Sciences, University of Aberdeen, Aberdeen, United Kingdom; 4 Department of Cell Biology, Faculty of Science, Charles University, Prague, Czech Republic; Geisel School of Medicine at Dartmouth, UNITED STATES OF AMERICA

## Abstract

The *doublesex and mab-3 related transcription factor 1* (*dmrt1*) plays a crucial role in metazoan sexual differentiation. This gene, or its paralogs, independently became triggers for sex determination several times, including in the tetraploid African clawed frog *Xenopus laevis*. To explore functional evolution of this gene, we generated knockout lines of each of two *dmrt1* homeologs in *X. laevis* and an ortholog in the closely related diploid Western clawed frog *X. tropicalis*. Our findings evidence sex-specific functional evolution following duplication by allotetraploidization in an ancestor of *X. laevis*. In females, *dmrt1* was essential for fertility and oogenesis in the *Xenopus* ancestor, but this important function was lost (subfunctionalized) in one *X. laevis* homeolog (*dmrt1.S*) after allotetraploidization. In males – in sharp contrast – *dmrt1* was not essential for fertility and spermatogenesis in the *Xenopus* ancestor, but this essentiality was acquired (neofunctionalized) in the other *X. laevis* homeolog (*dmrt1.L*) after allotetraploidization. Transcriptomic analysis of the mesonephros/gonad complex during sexual differentiation identifies distinctive patterns of dysregulation in male and female knockouts of *dmrt1.L* and *dmrt1.S* relative to same-sex wildtype siblings, including possible autocatalysis of *dmrt1.L* and activation of the female-determining gene *dm-w*. Previous work demonstrates that *dm-w* was recently derived from partial gene duplication of *dmrt1.S* – a gene that our analysis demonstrates is non-essential in both sexes. Thus, in *X. laevis*, a developmental system was pushed past a “tipping point” to a novel state where sexual differentiation is now orchestrated by a sex-specific duplicate of a dispensable gene.

## Author summary

The *doublesex- and Mab-3 Related Transcription Factor 1* (*dmrt1*) gene plays an important role in sexual differentiation in most metazoans – including humans, fruit flies, and worms. To better understand how function of this important gene changes over time, we used genetic engineering to disable its function in two frog species in the genus *Xenopus*, including one species that has two copies of *dmrt1* that each arose from genome duplication. We uncovered a strikingly distinct history of functional evolution of this gene in females and males. In females, this *dmrt1* was necessary for fertility and egg production in the *Xenopus* ancestor, but this essentiality was lost after genome duplication in one of the two copies of *dmrt1*. In males – sharp contrast –*dmrt1* was not necessary for fertility and sperm production, but this essentiality was acquired in the other copy of *dmrt1* after genome duplication. *Dmrt1* is most often associated with male-related functions, but our results provide the first known example (to our knowledge) where this gene is necessary for female fertility and egg production. Together this study offers a compelling and surprising example of how functional evolution can vary dramatically between the sexes.

## Introduction

### Dmrt1 and sexual differentiation

Primary sexual differentiation refers to the developmental process by which female and male adult gonads emerge from the same precursor tissues. Primary sexual differentiation involves several developmental milestones including (a) primordial germ cell specification, (b) germ cell commitment and migration to the developing gonad and (in some species) epigenetic resetting, (c) meiotic entry, (d) differentiation of germ cells, and (e) differentiation and maintenance of somatic tissues in the gonad, including supporting cells and hormone-secreting cells.

In metazoans, the *doublesex and mab-3*-*related transcription factor 1* (*dmrt1*) plays a key role in sexual differentiation [1–3], including roles in several of these milestones (S1 Text). *Dmrt1* is in the *dmrt* gene family, whose members contain a motif called a “DM domain” that chelates zinc, binds to DNA in a sequence-specific way, and resembles a zinc finger [1]. These transcription factors bind DNA as homodimers or heterodimers with other DM domain-containing proteins [4]. *Dmrt1* is capable of binding to its own promoter and those of other *dmrt* family genes, which suggests a capacity for auto- and cross-regulation that is probably modulated by other genes [5]. DM-domain-containing genes interact with the minor and major grooves of DNA [6] and transcriptional suppression or activation by *dmrt1* may be achieved by blocking or recruiting other transcription factors, respectively. *Dmrt1* is a pioneer transcription factor that is capable of influencing the accessibility of closed chromatin to other regulators [7]. During development, the effects of *Dmrt1* frequently are realized in conjunction with *SRY-Box Transcription Factor 9* (*Sox9*) to promote and maintain male gonadal function [8]; these phenotypes are often achieved in opposition to effects of *forkhead box L2* (*Foxl2*) [9,10]. In addition to regulating many aspects of sexual differentiation, genes in the *Dmrt* family are involved with somitogenesis and neural development [5].

In vertebrates, adult gonads of each sex – the ovaries and testes – both have cellular structures that are dedicated to gamete production: the follicle in females and the seminiferous tubule in males. Follicles and seminiferous tubules both contain three main cell types: germ cells – eggs (females) or sperm (males), supporting cells – granulosa (females) or Sertoli (males), and hormone-secreting cells – theca (females) or Leydig (males). But these cell types in each sex nonetheless differ in many ways, such as the number of germ cells they contain, with follicles containing one oocyte and seminiferous tubule containing many spermatogonia [11]. In many species, including mammals, developmental plasticity of primary sexual differentiation persists to adulthood and fully differentiated gonadal tissue can be genetically reprogrammed to transition to the other sexual identity [12,13].

A master regulator (“trigger”) of sexual differentiation is a genetic or environmental factor that directs development towards one or the other sex phenotype, thereby spurring primary (gonadal) and secondary (non-gonadal) sexual differentiation. These triggers typically operate after the embryo is partially formed but has the capacity to develop into either sex phenotype. Through evolution, genetic triggers may arise via changed function of conserved sex-related components, recruitment of novel non-sex-related genes, and elimination of ancestral sex-related genes. In vertebrates, evolution of the “usual suspects” [14] provides several examples of sex-related genes becoming the master regulator of sexual differentiation. These include members of the transforming growth factor beta (TGF-β) signaling pathway such as paralogs and diverged alleles of *anti-Mullerian hormone* (*amh*) [15,16] and *gonadal soma derived growth factor* (gdsf) [17], the *SRY*-related HMG-box 3 (*Sox3*) transcription factor [18], and *dmrt1* [19–21]. There are several examples where *dmrt1* or its paralog was recruited to become the master regulator of sexual differentiation. For example, (a) in birds sex-differences in allelic dosage of *dmrt1* (one allele in females, two in males) governs primary sexual differentiation [22], (b) in medaka fish a male-specific paralog of *dmrt1* (*dm-Y*) is the trigger for male sexual differentiation [21], and (c) in the frog *Xenopus laevis* a newly evolved trigger for female differentiation (*dm-w*) was formed via partial duplication of *dmrt1* [19,23].

### Functional evolution of dmrt1 and dm-w in African clawed frogs (Xenopus)

Comparative (multi-species) studies of African clawed frogs (*Xenopus*) have refined our understanding of the origin of *dm-w* and dynamic function of this gene and of *dmrt1*. *Dm-w* arose via partial duplication of one of two homeologs of *dmrt1* (*dmrt1.S*) that (along with the other homeolog – *dmrt1.L*) were formed by allotetraploidization of an ancestor of subgenus *Xenopus* about ~20 million years ago [24,25]. This duplication event captured the DM domain of *dmrt1.S*, but the *dm-w* promotor acquired novel regulatory elements and a portion of its 3’ coding region and the 3’ UTR originated from another source [26,27]. After its origin, *dm-w* was lost several times in different *Xenopus* species, and in several other *Xenopus* species where this gene persists, it does not function as the female-determining gene [23,24,28–30].

All *Xenopus* species except *X. tropicalis* are allopolyploid, meaning they evolved from hybridization and subsequent fusion of two ancestral genomes with lower ploidy levels. Allopolyploidization occurred multiple times in *Xenopus*, including several instances of sequential allopolyploidization; extant species are either diploid (one species), allotetraploid (17 species), allooctoploid (seven species), or allododecaploid (four species) [25,31]. Genome duplication occurs in these frogs when non-reduced eggs (diploid or triploid) from hybrid females are fertilized by sperm from each parental species in successive generations [reviewed in 32]. Consequently, female-specific (W-linked) genes such as *dm-w* are not duplicated by allopolyploidization, whereas autosomal (and pseudoautosomal) genes such as *dmrt1* are duplicated.

In *Xenopus* allopolyploids, each allopolyploid genome has a separate subgenome that is derived from one of the two lower ploidy-level ancestors. Homeologs in each subgenome generally do not recombine, and they therefore have distinctive evolutionary fates such as neofunctionalization, subfunctionalization, and pseudogenization. *Dm-w* is thought to be a negative regulator of *dmrt1* [19,33] and the stoichiometries of these gene products in these allopolyploid species is thus potentially relevant to sex determination. In species in the subgenus *Xenopus*, genus *Xenopus*, pseudogenization occurred much more frequently in *dmrt1* homeologs in the small (“S”) subgenome than the large (“L”) subgenome [24], which suggests that function of the ancestral *dmrt1.S* homeolog may have been non-essential. In transgenic *X. laevis* expressing a knockdown construct against the *dmrt1.L* homeolog, the number of germ cells was lower in both sexes compared to same-sex wildtype individuals, and the developing gonads of two of six genetic females expressing this knockdown construct also expressed markers associated with testes, suggesting masculinization or de-feminization [34].

### Goals

*Dmrt1* has a broadly conserved role in vertebrate sexual differentiation and a *dmrt1* paralog (*dm-w*) triggers female differentiation in the African clawed frog *Xenopus laevis*. These observations raise the questions of whether function of *dmrt1* varies among *Xenopus* species, and if so how. To explore these questions, we knocked out function of both homeologs of *dmrt1* in *X. laevis* (*dmrt1.L, dmrt1.S*) and the *dmrt1* ortholog in the diploid species *X. tropicalis*. We evaluated the consequences of loss of function mutation in each of these genes in terms of sexual differentiation, gonad morphology, and (in *X. laevis*) the transcriptome of the gonad during sexual differentiation. Our results evidence a profound link between *dmrt1* and sexual differentiation and fertility in both sexes, and paint a detailed picture of a dynamic and sex-specific sex-related history of functional evolution.

## Results

### Dmrt1 *knockout mutations in* X. laevis *and* X. tropicalis

We generated knockout lines for *dmrt1.L* and *dmrt1.S* in *X. laevis* and *dmrt1* in *X. tropicalis* using CRISPR/Cas9 (Figs A and B in S1 Text). For *X. laevis dmrt1.L*, a seven-base pair (bp) frameshift deletion was introduced into the coding region after the tenth codon, which changed the subsequent codon from a proline to an arginine, altered the reading frame, and introduced a premature stop codon downstream of this. For *X. laevis dmrt1.S*, a (different) seven-bp frameshift deletion was introduced within the 11^th^ codon, which changed this codon from an arginine to a proline, altered the reading frame, and led to a premature stop codon downstream of this. For *X. tropicalis dmrt1*, a one bp frameshift insertion was introduced into the coding region within the 26^th^ codon, which changed this codon from a leucine to an isoleucine, altered the reading frame, and led to a premature stop codon downstream of this. The length of wildtype versions of these proteins is 336 (*X. laevis dmrt1.L* and *dmrt1.S*) or 337 (*X. tropicalis dmrt1*) amino acids; because these frameshift and nonsense mutations occur very early in the coding region of each gene, we consider them all to be null mutations.

### Knockout phenotypes

We did not detect an abnormal phenotype in any individuals that were heterozygous for a null allele in either sex for *dmrt1.S* or *dmrt1.L* in *X. laevis* or for *dmrt1* in *X. tropicalis.* All *X. laevis* individuals that were heterozygous for a mutant allele (*dmrt1.S*: 5 females, 9 males; *dmrt1.L*: 2 females, 4 males) developed into fertile sex phenotypes that were consistent with the genetic sex as determined by amplification of a portion of the female-specific gene *dm-w*. For *X. laevis* and *X. tropicalis*, fertility of both sexes of heterozygotes with a null allele were confirmed with at least three intercrosses of different F1 individuals to generate homozygous (F2) null individuals. This suggests haplosufficiency for fertility of the wildtype *dmrt1.L* and *dmrt1.S* alleles in *X. laevis* and the wildtype *dmrt1* allele in *X. tropicalis*.

For individuals that were homozygous for a null allele, we did not detect sex reversal in either sex for *X. laevis*. An absence of sex reversal was confirmed in homozygous null male and female *X. laevis* with PCR assays of a portion of *dm-w* (*dmrt1.S*: 3 females, 11 males; *dmrt1.L*: 12 females, 5 males). Moreover, genetically female *X. laevis* knockouts of *dmrt1.S* developed into phenotypic females with typical sexually dimorphic adult characteristics such as larger body size than males, larger cloaca, and egg-filled oviducts confirmed upon dissection. Genetically female *X. laevis* knockouts of *dmrt1.L* also developed into phenotypic females with the expected larger body size compared to males, but with qualitatively smaller cloaca and abnormal gonadal differentiation discussed below. Genetically male *X. laevis* homozygous nulls of *dmrt1.S* developed into phenotypic males with typical sexually dimorphic adult characteristics such as smaller body size than females, smaller cloaca, and two testes with normal histology. Genetically male *dmrt1.L* homozygous null individuals developed into phenotypic males but with abnormal gonadal differentiation discussed below. One genetically male *dmrt1.L* homozygous null individual developed only one testis but the four others developed two. Fertility of male and female *X. laevis* that were homozygous null for *dmrt1.S* was confirmed by crossing these individuals to wildtypes. As discussed below, female and male *X. laevis* that were homozygous for *dmrt1.L* null alleles were sterile.

In *X. tropicalis*, *dmrt1* homozygous null individuals had two phenotypic sexes based on internal and external anatomy (6 phenotypic females, 2 phenotypic males). We suspect sex reversal also did not occur in *X. tropicalis* but were unable to confirm this due to a lack of sex-specific markers that would permit us to ascertain the genetic sex of these individuals. The phenotypic females had the expected larger body size that is characteristic of wildtype individuals, but with abnormal gonadal differentiation similar to that observed in *X. laevis* females that were homozygous null for *dmrt1.L* (discussed below). In *X. tropicalis* homozygous null *dmrt1* females were sterile. The phenotypically male homozygous null *X. tropicalis* both were smaller than the females, as is characteristic of wildtype males. Both *X. tropicalis* *dmrt1* knockout males had two testes that contained morphologically normal sperm. Fertility of these *X. tropicalis dmrt1* knockout males was not tested due to the small sample size, but we suspect these males were fertile based on their testis histology.

### Gonad histology

**Fig 1:**
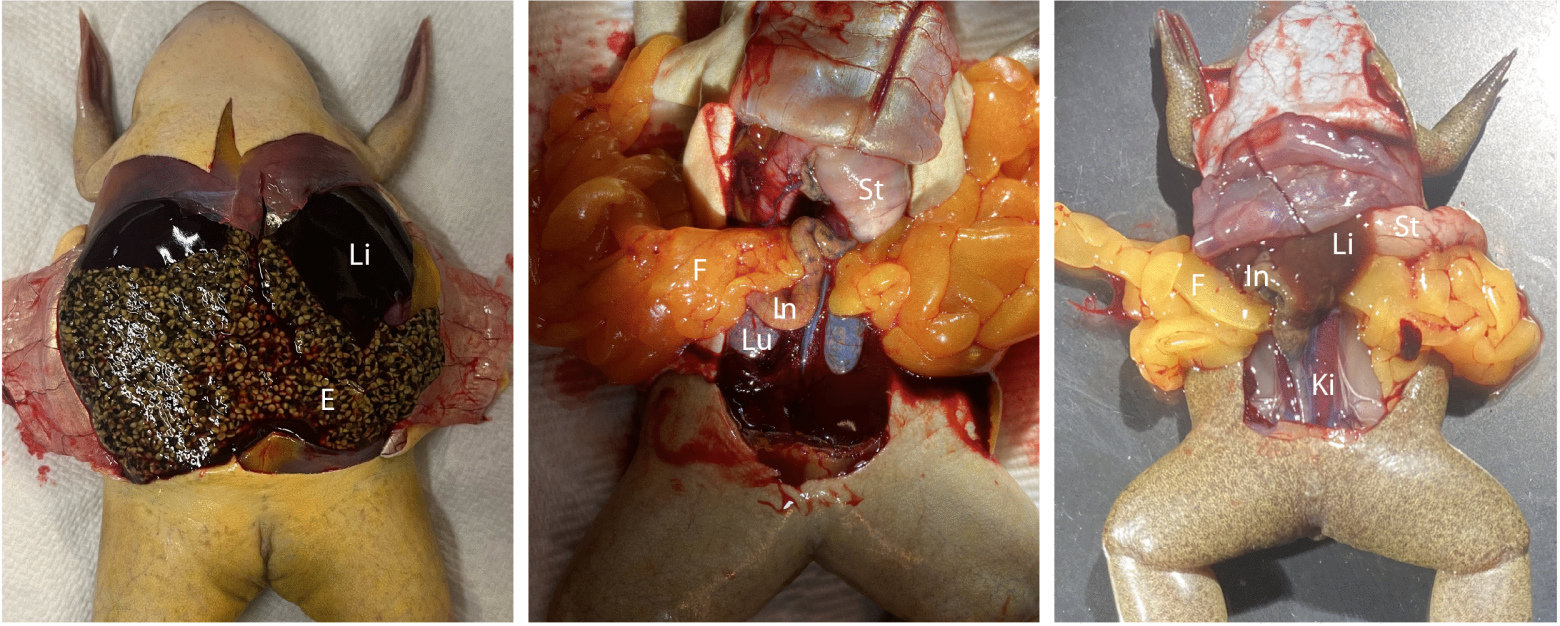
Dissected ventral views of a *X. laevis* wildtype female (left), and female knockouts for *dmrt1.L (X. laevis,* center) and *dmrt1* (*X. tropicalis, right).* The egg-filled oviduct (E) is visible in wildtype females but knockouts of *X. laevis dmrt1.L* and *X. tropicalis dmrt1* do not develop eggs; these animals have large fat bodies (F; orange-yellow structures), which are also present but much smaller in wildtype females (in the left, this obscured by the egg-filled oviduct). Other organs are labeled including the liver (Li), intestine (In), Lung (Lu), Stomach (St), and Kidney (Ki). Sample identification numbers are *X. laevis* wildtype: female5; *X. laevis* female knockout for *dmrt1.L*: female1; *X. tropicalis* female knockout for *dmrt1*: female1.

In adult wildtype females, oviducts full of eggs are readily observed upon dissection and ovulation is elicited by injection of human chorionic gonadotropin (HCG). Despite several attempts with HCG, we were unable to elicit ovulation in *X. laevis dmrt1.L* homozygous null females or *X. tropicalis* homozygous null females. We dissected 12 *X. laevis dmrt1.L* homozygous null adult females and six *X. tropicalis dmrt1* homozygous null adult phenotypic females. All of them were found to completely lack eggs, though oviducts were detected (Figs 1 and B in S1 Text). Anomalous tissue growths stemming from the ventral surface of the midline of the kidney (where the oviduct originates in wildtype individuals) were observed in two *X. laevis dmrt1.L* homozygous null adult females. All of the homozygous null females were otherwise normal in size and healthy based on large fat bodies.

**Fig 2:**
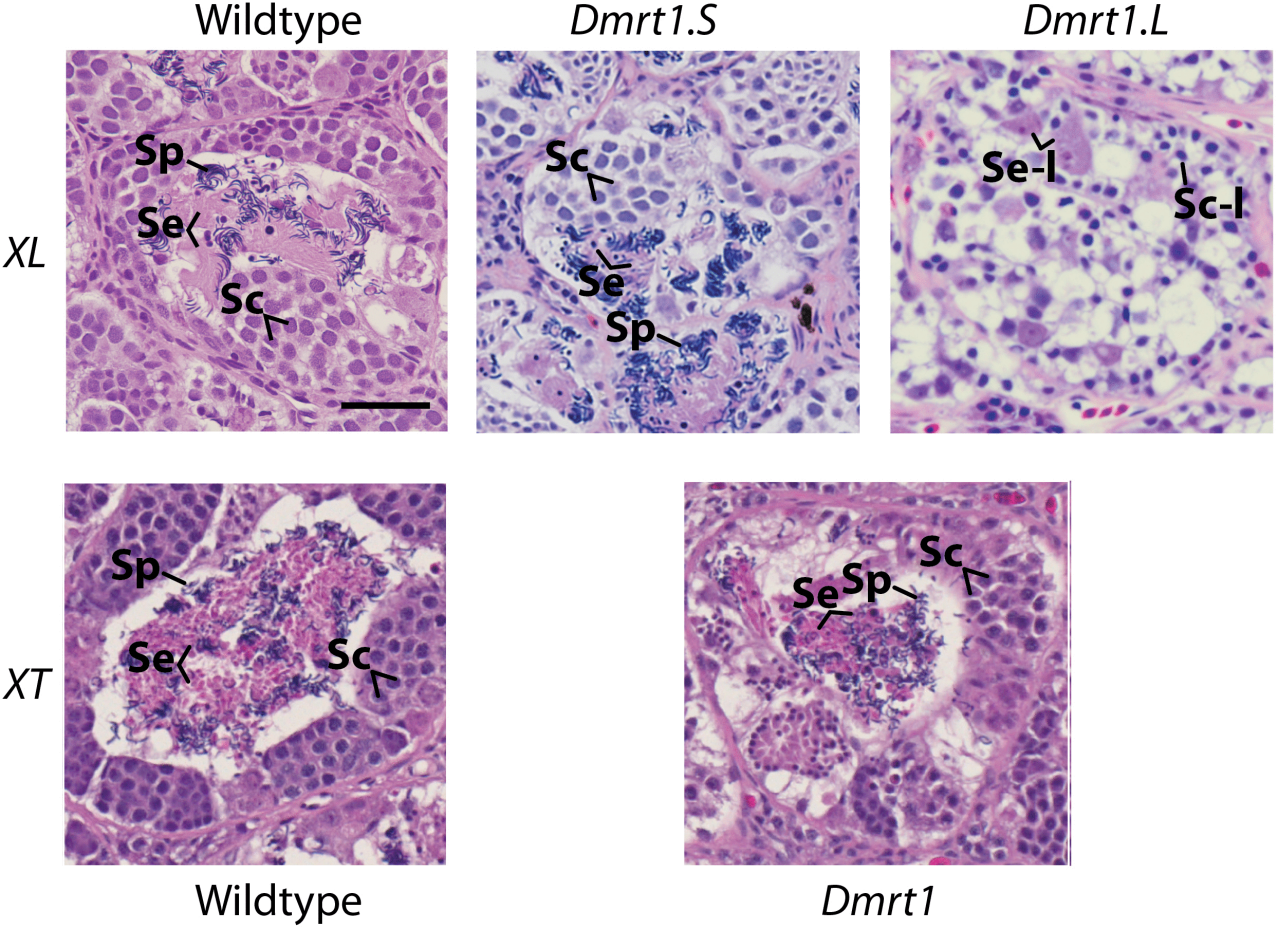
Testis histology of wildtype and knockout lines for *X. laevis* (*XL*) and *X. tropicalis* (*XT*) *dmrt1.L, dmrt1.S,* and *dmrt1.* The black bar in the upper left is 50 μm; spermatocytes (Sc), Sertoli cells (Se), and late spermatids (Sp) are labeled, except for *dmrt1.L* where spermatocyte-like (Sc-l) and Sertoli-like (Se-l) cells are labeled and spermatids are not present. Sample identification numbers are *X. laevis* wildtype: 1841; *X. tropicalis* wildtype: 1900; *X. laevis dmrt1.S*: 197A; *X. laevis dmrt1.L*: 1929; and *X. tropicalis dmrt1*: 1993. Scale bar is 50μm.

We performed histological analysis on (male) adult testes from three *X. laevis dmrt1.L* homozygous knockouts, six *X. laevis dmrt1.S* homozygous knockouts, two *X. tropicalis* homozygous knockouts, six wildtype *X. laevis*, and three wildtype *X. tropicalis.* Example sections are presented in Figs 2 and C in S1 Text. Seminiferous tubules were evident in all testes we examined. Qualitatively similar densities of mature spermatids were observed in the *X. laevis dmrt1.S* knockout individuals and in *X. laevis* wildtype individuals (and a quantitative analysis is discussed below), but no mature spermatids were observed in the histological preparations of *X. laevis dmrt1.L* knockout individuals (though a small number of morphologically abnormal sperm were detected using SEM, please see below). In the *X. laevis dmrt1.L* knockout individuals, we were able to identify Leydig-like cells on the periphery of the seminiferous tubules, and Sertoli-like cells within the seminiferous tubules (but lacking associated spermatids). Dark-staining nuclei from spermatocyte-like cells were apparent. One of the *X. laevis dmrt1.L* knockout males sampled had only one testis, though this also occasionally happens spontaneously in wildtype individuals. No anomalies were detected in testis histology of *X. tropicalis* homozygous null males compared to conspecific wildtypes.

Using digital images of our histological preparations, we quantified sperm abundance in the *X. laevis* wildtype and *dmrt1.S* knockouts and found no significant decrease in abundance in the knockouts compared to wildtypes (*P* = 0.93; one-tailed t-test). In fact, in the *X. laevis* wildtypes, mature spermatids comprised a lower proportion of the section (mean = 1.2%, standard error = 0.3) compared to *dmrt1S* knockouts (mean = 2.6%, standard error = 0.7), which is the opposite of our one-sided expectation that the knockouts would have a lower density of mature spermatids. In *X. tropicalis* we lacked biological replicates, but quantification of two testes from one *X. tropicalis* knockout had a similar proportion (mean = 1.9%, standard error = 0.6) to two testes from one wildtype individual (mean = 2.3%, standard error = 0.4); this difference was not significant (*P *= 0.33; one-tailed t-test). We also quantified sperm abundance in the *X. laevis dmrt1.L* heterozygotes and compared these to the wildtype abundances. We detected lower abundance in the *X. laevis dmrt1.L* heterozygotes (mean = 0.7%, standard error = 0.2) but this difference was not significant (*P* = 0.11; one-tailed t-test).

To further investigate the abundance of mature sperm in *dmrt1.L* null males, we examined live sperm in one mutant and one wildtype individual using a hemacytometer. A small number of sperm were detected that appeared to be mature (though not necessarily capable of insemination), but the abundance of sperm in one *dmrt1.L* null sample was substantially lower (~502 sperm/mg of testis tissue) than a wildtype male (~141,150 sperm/mg of testis tissue). Taking into account the weight difference between the two testes, the wildtype male had ~ 842 times more sperm than the *dmrt1.L* null male. We do not provide variances on these estimates from live sperm because the analysis was performed on only one individual of each genotype (knockout, wildtype). We then used scanning electron microscopy to visualize individual sperm from a wildtype and a homozygous null *dmrt1.L* male. The morphology of the sperm of homozygous null *dmrt1.L* male was distinctive, with the sperm heads were significantly shorter (mean = 17.17 μm, standard error = 1.15) than wildtype sperm (mean = 22.49 μm, standard error = 0.50; *P *= 0.003, one tailed t-test). The effect size [35] of this comparison is large (Cohen’s *d* = 2.55), and indicates that >99% of the sperm heads of a homozygous null *dmrt1.L* male would be smaller than the mean length of wildtype sperm heads. The mutant sperm heads were encased or associated with an unidentified matrix or tissue (Fig D in S1 Text), though we are unable to determine whether this was a technical artifact.

### Transcriptome analyses

We performed an analysis of differential expression in transcriptomes from bulk RNAseq data from mesonephros/gonad tissue at tadpole stage 50, which is the developmental stage where sexual differentiation is triggered by transient expression of *dm-w* (Yoshimoto et al., 2008). For each *X. laevis* knockout line (*dmrt1.S, dmrt1.L*), we used two approaches to compare transcriptomes of same-sex wildtype and knockout individuals (Analyses 1 and 2; see Methods). Importantly, comparisons within each line were among F2 siblings (including wildtype and homozygous null individuals) that were raised in the same tank under identical conditions. Significantly differentially expressed genes recovered from these analyses are provided in S1 and S2 Tables. As expected, expression of *dm-w* was detected in almost all females (e.g., Analysis 1 unnormalized median = 23.5; counts all were ≥ 7 except one individual with a count of zero) but no males (Analysis 1 unnormalized median = 0; all counts were ≤ 1).

When comparing *dmrt1.S* knockout females to wildtype females, the significantly differentially expressed genes were few in number and non-overlapping across the two analyses (Analysis 1: *n* = 20; Analysis 2: *n = *13; overlap: 0 genes). When comparing *dmrt1.S* knockout males to wildtype males, more differentially expressed genes were detected and these had extensive overlap across the two analyses (Analysis 1: *n* = 1251; Analysis 2: *n = *1373; overlap: 850 genes). There were many differentially expressed genes in the comparisons between female wildtype and *dmrt1.L* knockout mesonephros/gonads (Analysis 1: *n* = 337; Analysis 2: *n = *953; overlap: 303 genes). The comparison between wildtype males and *dmrt1.L* knockout mesonephros/gonads identified a small number of differentially expressed genes (Analysis 1: *n* = 5; Analysis 2: *n = *37; overlap: 4 genes). Taken together, these findings are consistent with distinctive effects of both knockouts on the transcriptomes of the developing mesonephros/gonad of each sex, with the most pronounced transcriptomic effect observed in the male *dmrt1.S* knockout and the female *dmrt1.L* knockout compared to same-sex wildtypes.

With an aim of understanding how and whether these transcriptomes were feminized or masculinized, we intersected the sets of differentially expressed transcripts with previously reported significantly sex-biased transcripts in wildtype *X. laevis* mesonephros/gonads at tadpole stage 50 [23]. These data were collected from wildtype individuals from each mutant line that were raised in the same tank under identical conditions. For Analysis 1, two (10%), 61 (5%), three (1%), and zero (0%) of the significantly differentially expressed transcripts in the *dmrt1.S* females, *dmrt1.S* males, *dmrt1.L* females, and *dmrt1.L* males, respectively, were also significantly sex-biased in at least one of three comparison between the developmental-staged matched mesonephros/gonad transcriptome of wildtype siblings. For Analysis 2, zero (0%), 65 (5%), seven (1%), and two (5%) of the significantly differentially expressed transcripts in the *dmrt1.S* females, *dmrt1.S* males, *dmrt1.L* females, and *dmrt1.L* males, respectively, were also significantly sex-biased in the developmental-staged matched mesonephros/gonad transcriptome of wildtype siblings.

Gene ontology analysis did not identify significant enrichment of gene function in differentially expressed transcripts in the *dmrt1.L* knockout in males, and only one cellular component enrichment (immunoglobulin complex) in the *dmrt1.S* knockout in females (S3 and S4 Tables); this is likely because relatively few differentially expressed genes were detected in these analyses. However, the other comparisons had diverse ontological enrichments. Notable ontological enrichment of differentially expressed genes in the *dmrt1.S* knockout transcriptome of males included genes involved with gamma-aminobutyric acid (GABA) biosynthesis and mitochondrial function. Notable ontological enrichment of differentially expressed genes in the *dmrt1.L* knockout in females included genes involved with sterol desaturase activity and the fatty acid elongase complex.

### Expression of sex-related genes

We also considered individual expression of 90 sex-related genes (Methods) by sex for each *X. laevis* knockout line. For Analysis 1 comparisons of female *dmrt1.S* knockout line to same-sex wildtypes, none of these sex-related genes were individually significantly differentially expressed (adjusted *P* value* *< 0.10). However, for this comparison with Analysis 2, *dmrt1.L* was individually significantly differentially expressed (lower in the knockout mesonephros/gonad). For Analysis 1 comparisons of male *dmrt1.S* knockout line to same-sex wildtypes, four of these 90 genes were individually significantly differentially expressed in males: *gata4.L* (female-related)*, nr5a1.L* (steroidogenic)*, ptgds.S* (male-related)*, inhbc.1.L* (testis differentiation). Each of these genes was more highly expressed in the male *dmrt1.S* knockout mesonephros/gonad compared to wildtype*.* For this comparison with Analysis 2, the same genes were significantly differentially expressed and *dmrt1.L* and *ddx25.L* were also differentially expressed (both were also higher in the knockout mesonephros/gonad). This suggests that *dmrt1.S* may play a role in inhibiting expression of several sex-related genes in males.

For Analysis 1 comparisons of female *dmrt1.L* knockout line to same-sex wildtypes, three of the 90 sex-related genes were significantly differentially expressed: *dmrt1.L* and *dm-w* (female-related), *cyp26b1.L* (limits expression of *stra8*)*.* The *dmrt1.L* and *dm-w* genes were more highly expressed in the female wildtype mesonephros/gonad, and *cyp26b1.L* was more highly expressed in the female *dmrt1.L* knockout mesonephros/gonad. For Analysis 2, these three genes were also significantly differentially expressed, as were the androgen receptor (higher in the female *dmrt1.L* knockout gonad) and vimentin (*vim.L*, higher in wildtype). For Analysis 1 and 2 comparisons of *dmrt1.L* male knockout line to same-sex wildtypes, one of 90 sex-related genes was individually significantly differentially expressed: *dnd1.L* (germ-cell specific); this gene was more highly expressed in the male *dmrt1.L* knockout mesonephros/gonad.

In *X. laevis*, knockout of the trigger for femaleness (*dm-w*) masculinizes the transcriptome of the female mesonephros/gonad at the developmental turning point of sexual differentiation [23]. We performed permutations to evaluate whether knockouts of *dmrt1.S* or *dmrt1.L* might have similar effects (Methods). When considering 90 sex-related genes (Methods) in females we had the expectation that knockouts could lead to masculinization and cause the female knockout: female wildtype expression ratio to be significantly more positively correlated with the wildtype male: wildtype female expression ratio as compared to random expression ratios drawn from the knockout and wildtype transcriptomes. In males we expected the opposite (i.e., we expected feminization and an atypically negative correlation). However, we recovered no significant support for these predictions (S5 Table). This is consistent with a pattern of dysregulation caused by these knockouts that is more complex than complete or partial sex reversal.

### Subgenome effects

One possibility is that each knockout gene disproportionately affected expression of other genes in the same subgenome. To evaluate this possibility, for each analysis, we calculated the proportions of differentially expressed genes in each subgenome. In Analysis 1, about 60% of the differentially expressed genes in each of the four comparisons were in the L subgenome, which closely matches the proportion of the transcriptome that is encoded by this subgenome [about 57% of the protein coding genes are in the L subgenome; [36]. Analysis 2 provided some support for subgenome bias based on a small sample: all (*n* = 13) differentially expressed genes recovered from the analysis of *dmrt1.S* females were in subgenome L and 80% of the differentially expressed genes recovered from the analysis of *dmrt1.L* males (*n *= 37) were in subgenome L. However, for Analysis 2, the other two comparisons both had about 60% of the differentially expressed genes being in subgenome L. Overall these results do not point to a strong or consistent effect of a knockout on expression of other genes in the same subgenome.

## Discussion

Primary sexual differentiation is a developmental turning point where a bipotential gonad becomes either an adult male or female gonadal structure [37]. In addition to other mechanisms, sexual differentiation can be initiated by genes that are found in only one sex, such as the female-specific gene *dm-w* in the frog *X. laevis*, which is derived from partial gene duplication of the sex-related gene *dmrt1* [19]. Depending on the presence or absence of a trigger for sex determination (be it genetic or environmental), sexual differentiation also involves sex-related autosomal (and pseudoautosomal) genes that are found in both sexes, such as (in many metazoans) *dmrt1*. In this study, we analyzed knockout lines of each homeolog of *dmrt1* (*dmrt1.L, dmrt1.S*) in the allotetraploid African clawed frog *X. laevis,* and a knockout line of the *dmrt1* ortholog in the diploid Western clawed frog *X. tropicalis*. Phenotypic and transcriptomic analysis of female and male individuals of these mutant lines provided a detailed perspective on sex-specific functional evolution of *dmrt1* after genome duplication in the ancestor of *X. laevis*. As discussed below, these findings highlight how rapid evolution of sex determination may be realized by modest functional transitions across biological tipping points – biological thresholds where a small genetic change leads to substantial developmental or phenotypic consequences.

### *Sex-specific functional evolution of dmrt1 following allotetraploidization in* Xenopus

**Fig 3:**
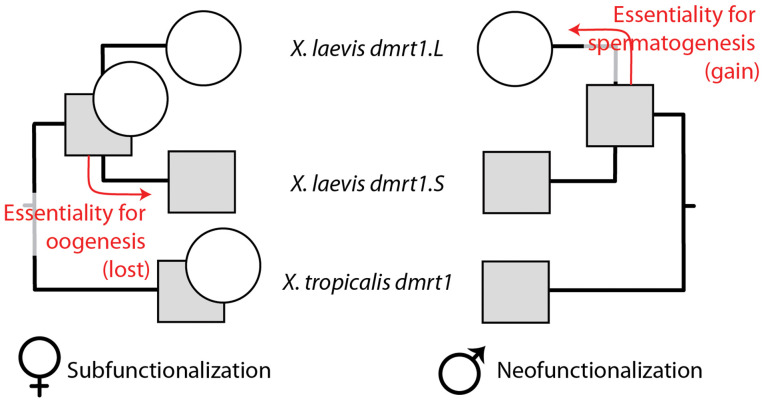
Sex-specific functional evolution of *dmrt1* following genome duplication in *X. laevis.* In females (left) subfunctionalization occurred when essentiality for female fertility, ovarian development, and oogenesis (circles) was lost in *dmrt1.S*; in males (right), neofunctionalization occurred when essentiality for male fertility and sperm (circles) was acquired in *dmrt1.L*. Squares indicate a non-essential subfunction that may partially overlap with the circles; based on patterns of expression divergence [34], this subfunction is probably mostly restricted to somatic cells in the male gonad.

In females, we infer the ancestral *dmrt1* protein to have been essential for ovarian development (Fig 3). This essentiality was retained in *X. tropicalis dmrt1* and in *X. laevis dmrt1.L* but lost in *dmrt1.S* following its origin by allotetraploidization in the ancestor of *X. laevis* ~20 million years ago [25,36]. Loss of female-related ancestral function in *dmrt1.S* is evidenced by the relatively low number of differentially expressed genes in the female *dmrt1.S* knockout mesonephros/gonad (S1 and S2 Tables) and by the primarily male-specific expression domain of this gene [34]. In sharp contrast, female *dmrt1.L* and male *dmrt1.S* knockout transcriptomes in the developing mesonephros/gonad both have extensive expression divergence compared to the same-sex wildtype transcriptome. Together this argues for subfunctionalization, wherein *dmrt1.L* and *dmrt1.S* both have distinctive (but potentially partially overlapping) biological functions, with a major female-related ancestral function of *dmrt1* – essentiality for ovarian development – having been lost in *dmrt1.S*.

In males, we infer the ancestral *dmrt1* protein to not have been essential for sperm development because *X. laevis dmrt1.S* knockout males and *X. tropicalis dmrt1* knockout males are viable and fertile. Essentiality for normal sperm was acquired by *X. laevis dmrt1.L* after genome duplication in the ancestor of *X. laevis* is demonstrated by the *dmrt1.L* knockout phenotype in males which includes infertility, abnormal testis histology, massively reduced sperm count, and anomalous sperm morphology (Figs 2 and D in S1 Text). This is consistent with neofunctionalization in males that occurred in *dmrt1.L* following allotetraploidization in the ancestor of *X. laevis*. Taken together, interpretation of these knockout phenotypes in a phylogenetic context evidences sex-specific functional evolution (female subfunctionalization, male neofunctionalization) following allopolyploidization in *Xenopus*.

*Dmrt1* is postulated to play a role in oogenesis in several other species, such as the Japanese eel (*Anguilla japonica*) and Atlantic cod (*Gadus morhua*) [38,39]. In female mice (*Mus musculus*), *dmrt1* knockout individuals are fertile, but compared to wildtypes, they have a reduced number of primordial follicles in the juvenile ovary, reduced expression of *stra8* – a meiotic inducer that is normally activated by *dmrt1* in females [40], and impaired meiotic initiation [40]. In zebrafish (*Danio rerio*), *dmrt1* is linked to oogenesis because in males it represses *foxl2*, which is involved in ovarian development [41]. Although *dmrt1* is implicated in ovarian development in several species, to our knowledge, *X. laevis* and *X. tropicalis* are the only examples thus far where *dmrt1* knockout completely prevents oogenesis.

A lack of sex reversal in *Xenopus* male *dmrt1* knockout lines contrasts with findings from several other species. For example, in Nile tilapia fish (*Oreochromis niloticus*), knockout of *dmrt1* causes male-to-female sex reversal, whereas females remain unaffected [9]. Likewise, in zebrafish knockout of *dmrt1* causes male-to-female sex reversal [41], and male (ZZ) chickens (*Gallus gallus domesticus*) that carry only one allele of *dmrt1* develop ovaries rather than testes [22]. However, in humans, deletion of one allele of *dmrt1* leads to male-to-female sex reversal but with gonad dysgenesis, including the formation of Leydig-like cells [42]. When *dmrt1* is disrupted in tilapia using transcription activator-like effector nucleases (TALENs), most male germ cells do not develop past the stage of secondary spermatocytes, but sex reversal is not observed [43]. Likewise, in Japanese eel, *dmrt1* is highly expressed in spermatogonia B cells, spermatocytes, and spermatids, but not in Sertoli cells, spermatozoa, or spermatogonia A cells [which are the precursors of spermatogonia B cells; 38]. This suggests that *dmrt1* may be involved in the developmental progression from spermatogonia B cells to mature spermatids in this species. Thus, the role of *dmrt1* in Japanese eel may be similar in some ways to that of *dmrt1.L* in *X. laevis*.

### *Functional divergence following allotetraploidization in* Xenopus

The *dmrt1.L* gene in *X. laevis* and the *dmrt1* gene in *X. tropicalis* both have two transcription initiation sites that determine inclusion of a noncoding first exon that is not present in the *dmrt1.S* gene in *X. laevis* [27,34]. The translation start site of these isoforms is in the second exon, so in each species the peptide encoded by the two *dmrt1.L* transcripts is presumably the same. However, the expression domains of the two *dmrt1.L* isoforms differs substantially in *X. laevis*: in adult males, the longer isoform (that includes the noncoding first exon) is more highly expressed in germ cells, and the shorter isoform is more highly expressed in somatic tissue [34]. Nonetheless, expression of the shorter isoform of *dmrt1.L* in somatic gonadal tissue is still lower than expression of *dmrt1.S* [34]. In gonads of tadpole stage 65 and in adults, the longer isoform of *dmrt1.L* is expressed primarily in germ cells of both sexes, whereas *dmrt1.S* is expressed exclusively in somatic tissue of male (but not female) gonads [34]. This divergent expression suggests an important role of *dmrt1.L* in germ cell development in both sexes, and of *dmrt1.S* in somatic (Sertoli, Leydig) cell function in males. Co-expression of *dmrt1.L* and *dmrt1.S* in somatic gonadal tissue of males – albeit at different levels – suggests that these genes may have overlapping functions in this tissue type. Interestingly, immunofluorescence demonstrates that the female-determining *dm-w* protein co-localizes with *dmrt1* (presumably encoded by the *dmrt1.L* gene because *dmrt1.S* is not highly expressed in females) in somatic but not germline tissues of the developing gonad [33].

These divergent expression domains are highly consistent with the knockout phenotypes of *dmrt1.L* (i.e., the absence of germ cells in both sexes), and with the extensive expression divergence of the male *dmrt1.S* knockout mesonephros/gonad transcriptome relative to same sex wildtypes (1251 or 1373 differentially expressed genes depending on the analysis, S1 and S2 Tables). In males, Sertoli cells support fundamental aspects of spermatogenesis by supplying nutrients to developing germ cells, regulating cell cholesterol levels, and secreting androgen-binding protein which assists with the uptake of testosterone produced by Leydig cells [44,45]. Expression divergence of the female *dmrt1.S* knockout mesonephros/gonad transcriptome relative to same sex wildtypes is comparatively modest (S1 and S2 Tables) – this is perhaps unsurprising since *dmrt1.S* is not highly expressed in the female mesonephros/gonad at this stage of development [34].

Gene ontogeny analysis indicates that the transcriptome of the developing mesonephros/gonad of *dmrt1.L* knockout in females is enriched for differentially expressed genes involved in the sterol biosynthesis and the fatty acid elongase complex (S3 and S4 Tables) In females, follicular fluid-derived meiosis-activating sterol may contribute to oocyte maturation [46]; fatty acids play key roles in oogenesis as metabolic substrates and as precursors to cell signaling molecules [reviewed in 47]. The absence of oocytes coupled with differential expression of these key biosynthetic pathways in *dmrt1.L* knockouts suggests a biological connection between these transcriptome-level and tissue-level phenotypes in *Xenopus*.

In *X. laevis* tadpole stage 50 mesonephros/gonads, *dmrt1.S* is lowly expressed [this study; 34]. However, the tadpole stage 50 mesonephros/gonads transcriptome of the male homozygous *dmrt1.S* knockout line is substantially distinct from same-sex wildtype transcriptomes, which suggests that *dmrt1.S* influences expression of other genes in males prior to tadpole stage 50. This is interesting from the standpoint of *dm-w* expression, which is thought to initiate feminization of an otherwise undifferentiated gonad at tadpole stage 50 [19], raising the question of how expression of *dmrt1.S* prior to gonadal differentiation could have a male-specific effect. One possibility is that the hallmarks of tadpole stage 50 that we used (Methods) are not precisely or consistently correlated with gonadal development, which could have already been initiated. Alternatively, it is conceivable that *dm-w* could have a role in initiating sexual differentiation at an even earlier developmental stage, even though expression was not detected in tadpole mesonephros/gonads at stage 48 using quantitative PCR [19].

The *dmrt1.S* knockout transcriptome of males had enrichments in genes related to GABA biosynthesis and mitochondrial function. GABA is a non-protein amino acid that is an important neurotransmitter whose activity is mediated through three receptors [48]. Interestingly, one of these receptors – GABA_A_ – is negatively modulated by neuroactive steroids such as β-estradiol and pregnenolone sulfate [49]. Mitochondria are essential sites for steroid synthesis in steroidogenic cells in the gonads and other tissues [50] and play crucial roles in sperm maturation and function [reviewed in 51]. Collectively, these enrichments point a role of *dmrt1.S* in steroid-related function even though the *dmrt1.S* knockouts of both sexes are fertile. It is conceivable that some of these functions of *dmrt1.S* also are carried out by *dmrt1.L* which is co-expressed in somatic tissues, albeit at lower levels [34]. Moreover, pseudogenization of homeologs of *dmrt1.S* probably occurred many times independently in different *Xenopus* species [24], which further highlights the non-essential nature of this locus.

*Dmrt1.L* and *dm-w* were both significantly more highly expressed in the female wildtype mesonephros/gonad compared to the *dmrt1.L* female knockout. This is consistent with the possibility that *dmrt1.L* positively regulates its own expression [5], and also the expression of *dm-w*. The DNA-binding (DM) domain of *dmrt1* and *dm-w* are almost identical at the peptide level [19] but these genes have different 5’ regions that contain different promoters (TATA-less and TATA-containing, respectively; [27]). It is conceivable that the DM-domain of *dmrt1.L* can interact with both of these promoters, or that *dmrt1.L* interacts with some other regulatory region shared by these paralogous genes.

Sex-specific expression of sex-related autosomal genes frequently drives sexual differentiation, such as sex-biased regulation of the *androgen receptor* in humans [52]. Perhaps less appreciated (but demonstrated here) is that the same gene can be expressed in both sexes but with profoundly distinctive effects and with a sex-specific history of functional evolution. Mechanistically, this could be realized via sex-specific expression domains or – for transcription factors – sex-differences in chromatin landscape and other co-factors that influence activation and repression of other genes. These possibilities are promising directions for future study aimed at understanding sex-differences in functional evolution of key genes such as *dmrt1*.

### Tipping points and rapid evolution of sex determination

Another implication of this study relates to a central question in macroevolutionary biology that asks whether evolution generally proceeds via small (phyletic gradualism) or large (punctuated evolution) steps [53]. An analogous (and similarly qualitative) question asks whether the genetic architecture of key developmental milestones (such as sexual differentiation) evolve through large or small functional changes [54,55]. Moreover, mutational robustness of biological systems may be achieved through genetic redundancy (e.g., duplicated genes, pathways), feedback mechanisms, haplosufficiency, and molecular chaperones that help other proteins fold properly, even when they possess destabilizing mutations [56]. The tempo of evolution (slow and steady versus bursts of change interceded by periods of stasis) likely depends on the genetic architecture that underpins a phenotype, including the mutational target sizes of pathway components that are dosage sensitive or haploinsufficient. These components could have large effects on the nature of phenotypic evolution.

At one extreme, sexual differentiation could be governed by tipping points in gene expression or even be essentially random [57]. Tipping points are a key component of environmental sex-determination, for example when incubation temperature being above or below a threshold temperature determines whether an embryo develops into a female or male individual [e.g., 58]. Tipping points are also relevant to sex chromosome evolution, such as when some threshold of divergence between sex chromosomes hinders future sex chromosome turnover [59] or when a threshold amount of weakly deleterious mutations have accumulated on the Y chromosome make it favorable to silence the entire Y [60]. More generally, triggers for sex determination could evolve rapidly but via small genetic changes in “peripheral” genes, if these genes then impinge on a conserved core developmental system that orchestrates sexual differentiation [61].

On the other extreme, sex determination may involve large disruptive genetic changes characterized by novel genetic interactions that abruptly shift an entire developmental trajectory to a new state. For example, some triggers for sex determination arise through allelic divergence of key sex-related genes [*Sry* in therian mammals; 62] or haploinsufficiency [*dmrt1* in birds; [20], which presumably had functional consequences for core interactions of the genetic network underpinning sexual differentiation. Rapid evolution of core components of the sexual differentiation cascade could be achieved if the origin of sex-specific and developmental-stage-specific expression of a new genetic trigger mitigates pleiotropic constraints present in the ancestral gene (for triggers that arise from gene duplication) or allele (for triggers that arise from allelic divergence) [63].

In different frog species in the genus *Xenopus*, several novel triggers for sex determination appeared independently [29], including *dm-w*, which is a partial gene duplicate of *dmrt1.S* [19, 24]. Results from knockout experiments presented here demonstrate that the ancestral gene (*dmrt1.S*) of *dm-w* was non-essential, in the sense that female and male individuals carrying homozygous knockouts of *dmrt1.S* are viable and fertile. Non-essentiality of *dmrt1.S* is further supported by pervasive pseudogenization of *dmrt1.S* following genome duplication in several *Xenopus* species [24]. Non-essentiality of *dmrt1.S* is presumably facilitated by overlapping expression and compensatory function of *dmrt1.L* in somatic cells of the gonad [34]. In this way, a new trigger for sex determination (*dm-w*) arose by partial gene duplication of a core gene within the sexual differentiation pathway (*dmrt1.S*), but this core gene had probably already become largely dispensable as a consequence of subfunctionalization. Subfunctionalization involved the origin of a male-specific and somatic cell-specific expression domain of *dmrt1.S* in the developing gonad. Partial duplication of this dispensable gene gave rise to *dm-w* – an allele whose expression was also somatic cell-specific [33], but which feminized the developing gonad, and therefore had female-specific expression. This event pushed an entire developmental system past a genetic tipping point to a new state where sex determination of *X. laevis* was then orchestrated by a new (and highly influential) gene (*dm-w*).

## Methods

### Ethics statement

All work with live animals was approved by the Animal Use Committee at McMaster University (AUP# 17–12–43) and the Institutional Animal Care and Use Committee at the Marine Biological Laboratory (IACUC # 22–29).

### Knockout lines

We used CRISPR-Cas9 [64] to introduce deletions and frameshift mutations in the 5’ portion of the coding regions of *dmrt1.S* and *dmrt1.L* in *X. laevis* and *dmrt1* in *X. tropicalis*. Single stranded RNA guides were generated from a DNA template that contained an SP6 promoter sequence and a universal reverse primer for transcription using the Megascript SP6 transcription kit (Life Technologies, USA). Sequences of guides and primers for genotyping are itemized in S6 Table.

Single-stranded guide RNA was then injected with the Cas9 protein into one-cell embryos from *X. laevis* J-strain individuals and an inbred *X. tropicalis* line. The resulting F0 mosaic individuals were raised to sexual maturity and crossed with wildtypes to generate non-mosaic F1 individuals with germline transmission. F1s were then intercrossed to generate homozygous null and heterozygous F2 individuals for each locus (Fig A in S1 Text). Genotypes were determined by Sanger sequencing of PCR products amplified from DNA extracted from samples of foot webbing from using the DNeasy kit (Qiagen, Germany) following the manufacturer’s protocol.

To ascertain the genetic sex of each individual in our two mutant lines for *X. laevis*, we used four independent PCR amplifications, each with a different pair of primers that targeted portions of the coding region of exon 2 of the female determining gene *dm-w* [19] and three different portions of the female-specific 5’ upstream untranslated region of this gene: dmw_5pr_for_71 & dmw_5pr_rev_810, dmw_5pr_for_2762 & dmw_5pr_rev_3122, dmw_5pr_for_1300 & dmw_5pr_rev_2131 [24], and dmw_intron1_for1 & dmw_intron2_rev1 [23]. Independent successful amplification of each of these four regions identified genetic females and unsuccessful amplifications of each of these four regions identified genetic males; wildtype females were amplified in tandem as a positive control.

### Internal anatomy and histology

Wildtype and mutant frogs were dissected for anatomical and histological analysis from each of the knockout lines (S7 Table). In total, 45 *X. laevis* frogs were dissected including four wildtype females, 12 *dmrt1.L* homozygous null females, three *dmrt1.S* homozygous null females, eight wildtype males, five *dmrt1.L* homozygous null males, six *dmrt1.S* homozygous null males, two *dmrt1.L* heterozygous males, five *dmrt1.S* heterozygous males. Except for two *X. laevis* wildtype individuals, all other individuals (wildtype, heterozygous, homozygous) from each mutant line were F2 siblings that were raised in the same tank as the other individuals from each line. For the *X. tropicalis* line, a sex-specific marker is unavailable, so we instead evaluated the phenotypic sex of mutants based on dissection after euthanasia. In total, 13 *X. tropicalis* frogs were dissected including one wildtype female, seven *dmrt1* homozygous null females, three wildtype males, and two *dmrt1* homozygous null males.

To prepare tissues for histology, cardiac perfusion was performed using phosphate-buffered saline (PBS) with pH of 7.4, followed by fixation by perfusion with 10% formalin. PBS was injected into the bottom of the heart after clipping of the veinous vessels of the heart, until the fluid exiting these vessels was clear, usually around 25 ml. Next, approximately 25 ml of the formalin was injected into the bottom of the heart as a first step towards fixation of tissues. Following the perfusion, testes were dissected and fixed in 10% formalin for minimum of 48 hours, then were transferred to 70% ethanol for a minimum of 48 hours before histological analysis. Tissues were then embedded in paraffin, sectioned, and stained at the core histology facility at the McMaster Immunology Research Centre (Hamilton, Ontario). Four µm sections were stained with hematoxylin and eosin stain following the protocol recommended by Leica Biosystems for use with Leica’s SelecTech stains Hematoxylin 260MX, Eosin 515LT on the Leica Autostainer XL. Slides were imaged using the ZIESS Axioscan 7 slide scanner (10X; 0.45 NA). Cell types were evaluated following [65].

### Scanning electron microscopy

We used scanning electron microscopy (SEM) to visualize sperm of a wildtype and a *dmrt1.L* knockout male. Following euthanasia, testes were dissected, sliced into thin (~1mm) sections, and placed in 500 ul 2% glutaraldehyde for 2 hours. This mixture was then spun at 3,000 rpm for 30 seconds in a tabletop centrifuge and the supernatant was removed and discarded. Then 500ul of double distilled water was added and the tube was gently inverted. One drop of this solution and several 0.2X dilutions were dropped on coverslips and left to dry at room temperature. Coverslips were mounted onto SEM stubs with double-sided carbon tape, coated with approximately 15 nm gold in an Edwards S150B sputter coater, and then viewed in a Tescan Vega II LSU scanning electron microscope (Tescan USA, PA) operating at 20kV.

### Quantification of sperm

To better understand infertility of *X. laevis dmrt1.L* null males, we quantified concentrations of live sperm from these individuals. Testes were dissected from a *dmrt1.L* null male and a wildtype male, and each was then masticated in 100µl of 1.2x Marc’s Modified Ringer solution (MMR) prepared following Shaidani et al. [66]. For the *dmrt1.L* null sample, 10µl of this solution was diluted to 20 µl with the addition of 1.2x MMR. For the wildtype sample, 1µl of this solution was diluted to 99µl with the addition of 1.2x MMR. Three additional dilutions were then performed for each sample, and sperm cells were counted using a hemacytometer, and the total concentration of sperm was then calculated for each sample relative to the mass of testis tissue.

For *X. laevis dmrt1.S* knockouts (one testis from each of three individuals), *X. laevis dmrt1.L* heterozygotes (four testes from two individuals), and *X. tropicalis* knockouts (two testes from one individual), we also quantified the proportion of sperm in images of histological sections and compared these to wildtype individuals (*X. laevis,* one testis from each of three individuals; *X. tropicalis,* two testes from one individual). This analysis was not performed on *X. laevis dmrt1.L* knockouts because no mature spermatids were observed in histological sections from this line. We used GIMP version 2.10.38 (https://www.gimp.org/) to label sperm and then quantify the proportion of the section that was labeled (i.e., the number of pixels that were sperm divided by the number of pixels that were any testis tissue). Because sperm stains with similar color and intensity as other cellular structures, we manually removed off target labeling from each image to ensure accurate quantification. For each species, sperm abundance of each experimental genotype was compared to conspecific wildtypes using a one-sided t-test assuming equal variance.

### Fertility

Fertility was assessed for *X. laevis dmrt1.L* and *dmrt1.S* knockout males and females using in vitro fertilization (IVF) following the protocol of Shaidani et al. [66]. For each mutant line, we attempted to generate embryos *in vitro* using a wildtype male with a homozygous null female, or a wildtype female with a homozygous null male. For each fertility assay, we concurrently performed a cross between a wildtype pair using the same solutions and wildtype gametes (eggs or sperm depending on the assay) as a control. Fertilization was inferred to be successful if multiple cleavage events were observed. In total, the fertility assay was performed on three *dmrt1.L* knockout females, one *dmrt1.S* knockout female, two *dmrt1.L* knockout males and three *dmrt1.S* knockout males.

### Transcriptome analysis of F2 progeny

Bulk transcriptome sequencing (RNAseq) data was performed on dissected mesonephros/gonad tissue from *X. laevis* F2 tadpoles at stage 50, which is when the gonads begin sexual differentiation (Yoshimoto et al., 2008). Tadpole stage 50 was determined based on morphological features of the tentacles and rear limb buds [67,68]. In total, this analysis consisted of 42 tadpoles. From the *dmrt1.L* line this included five wildtype males, three wildtype females, six null males, and six null females. From the *dmrt1.S* line, this included six wildtype males, six wildtype females, three null males, and seven null females. All individuals within each line (wildtype and null) were F2 siblings that were raised in the same tank under identical conditions. Sequencing of the *dmrt1.S* mutant line was performed in two separate runs. The first run included four knockout females, one knockout male, three wildtype females, and three wildtype males. The second run included three knockout females, two knockout males, three wildtype females, and three wildtype males. Procedures for RNA quality assessment, library preparation, paired-end sequencing, and read trimming are described in Cauret et al. [23]. Prior to analyses of differential expression, genes with an average of less than two reads per individual were removed.

We used two approaches to analyze the RNAseq expression data, and generally focus our interpretations on results that were consistent between these approaches. For the first approach (Analysis 1), normalized counts were obtained with STAR version 2.7.9a (Dobin et al., 2013) by mapping to the *X. laevis* version 10.1 genome assembly which was obtained from Xenbase [69]. Counts were then analyzed using EdgeR version 3.40.0 (Chen et al. 2016, McCarthy et al. 2012, Robinson et al. 2010), and for *dmrt1.S*, the lane effects were controlled for by including this variable in the design. For the second approach (Analysis 2) we obtained pseudocount data using Kallisto version 0.46.1 [70] and these data were then analyzed using DeSeq2 version 1.34.0 [71]. Using these two approaches, separate analyses were performed each mutant line (*dmrt1.L* or *dmrt1.S*) that compared mutant to same-sex wildtype individuals. Significantly differentially expressed genes were classified as those with a false detection rate (FDR) less than 0.10.

To further characterize the function of differentially expressed genes, a gene ontology (GO) analysis was completed on the set of genes that were significantly up- or down-regulated in each knockout line relative to same-sex wildtypes. Because many transcripts of *X. laevis* are not annotated, we relied on putative orthologous annotations from the human transcriptome GRCh38.p13 release 42 (Frankish et al. 2021). This was completed by using the discontiguous Mega blast algorithm to obtain annotations for each differentially expressed gene using the original gene sequences and estimating putative orthologs based on the best bit score within BLAST [72]. From here, the GO analysis was completed using a false discovery rate of 0.05 through Fisher’s exact test using the Gene Ontology Resource (http://geneontology.org/).

### Analysis of sex-related genes

As a complement to the gene ontology analysis of differentially expressed genes, an additional analysis that focuses on a set of 90 sex-related genes was also performed (S8 Table). These included (some with two homeologs): 74 genes that were previously identified be involved with female and male sex determination, steroidogenesis, and gonadal development in *Xenopus* [34,73,74]; *ddx25, dnd1, nanos1, spire1*, and *cyp17a1*, which are highly expressed in germ cells [34,74,75]; *sox9*, which is highly expressed in Sertoli cells and oocytes [76–78]; cytokeratin, which is expressed in immature Sertoli cells [78]; vimentin, which is expressed in immature and mature Sertoli cells [78]; *stra8*, which is activated by *dmrt1* in females and repressed by *dmrt1* in males [40,79]; *cyp26b1*, which limits expression of *stra8* in males [80]; *Xenopus* vasa-like gene 1 (*xvlg1*; a.k.a. *ddx4*), which is expressed in or essential for germ cells [81,82]; *gata1* which is expressed in mature Sertoli cells [76]; *cyp19a1, cyp17a1*, *sox9*, *gata4*, and *foxl2* which are more highly expressed in somatic compared to germ cells in female and male gonads [34,74,83–85].

For these 90 genes, expression ratios were determined for wildtype males:wildtype females and knockout females:wildtype females or knockout males:wildtype males within three different clutches (one each from the *dmrt1.L* and *dmrt1.S* lines and a third from a separate line that did not include immediate kin of any of the mutant individuals). For these 90 genes, the correlation between the female:male expression ratios and the null:wildtype expression ratio for each sex and each mutant line was assessed. For this analysis, no filtering was performed based on transcript abundance, but we did excluded outliers, defined as 1.5 times the interquartile range above or below the upper or lower quartile [23]. Spearman’s correlation was calculated between the non-outlier log2 fold changes [23]. A permutation test with 1000 replications was used to assess whether the observed correlation departed significantly from random expectations based on correlations between 90 randomly selected genes, following Cauret et al. [23]. In females, if knockouts lead to masculinization of the transcriptome, we expected the female knockout: female wildtype expression ratios to be significantly more positively correlated with the wildtype male: wildtype female expression ratios as compared to correlations between expression ratios of an equivalent number of random genes drawn from the knockout and wildtype transcriptomes. In males, if knockouts lead to feminization of the transcriptome, we expected the opposite – an atypically negative correlation between the male knockout: male wildtype expression ratios and the wildtype male: wildtype female expression ratios.

## Supporting information

S1 Text Functional roles of *Dmrt1* during vertebrate development. Fig A. (A) Sanger sequences of wildtype (wt) and homozygous knockout (ko) individual of *X. tropicalis* (top) and *X. laevis* (middle, bottom) illustrate loss of function frameshift mutations including a 1 bp deletion in *X. tropicalis*, and two independent 7 bp deletions in *X. laevis dmrt1.L* and *dmrt1.S*. Each mutation is in the coding region and very near the start codon (by interrupting the 26^th^, 10^th^, or 11^th^ amino acid out of 337 or 336 in total; see main text). (B) Distributions of exons (gray boxes), introns and flanking non-transcribed regions (black lines), the DM domain (black boxes in exons), and locations of frameshift mutations (red x followed by the first amino acid position that is affected by the mutation). Starts of transcription are indicated with black arrows, including both isoforms of *dmrt1.L*; start and stop of translation are indicated with a green arrow and “STOP” respectively. The number below exon indicate the number of amino acids encoded in wildtypes. Fig B. Empty oviducts (O) associated with the dissected of the kidney (K) and fat bodies (F) an *X. laevis dmrt1.L* homozygous knockout female. The ventral surface of the kidney is shown; anterior is on the top of the image. Fig C. Additional examples of testis morphology with labeling and black scale bar following Fig 2, including a wildtype individual (top: individual 185E) and a *dmrt1.L* homozygous knockout (bottom: individual 1880). A dotted yellow line in each image demarcates a seminiferous tubule. Scale bar is 50μm. Fig D. Scanning electron microscopy images of wildtype sperm (top left) and sperm from *X. laevis* homozygous knockout for *dmrt1.L*. Scale bars are 5μm.
(DOCX)

S1 Table Differentially expressed genes in same sex comparisons between knockout and wildtype tadpole gonads for X. laevis dmrt1. S and dmrt1.L lines using counts from STAR and analysis with EdgeR (Analysis 1). The four analyses performed include comparisons of female or male dmrt1S knockouts to same-sex wildtypes (dmrt1.S females, dmrt1.S males, respectively) and comparisons of female or male dmrt1.L knockouts to same-sex wildtypes (dmrt1.L females, dmrt1.L males, respectively). Negative log2 fold change (logFC) means expression is higher in the wildtype compared to the knockout; positive values indicate the opposite. Uncorrected (PValue) and false detection rate corrected (FDR) p-values are provided. Other information includes the chromosome and subgenome for each gene, the gene acronym for Xenopus (Xen_acro) and humans (Hum_acro), and whether each differentially expressed gene was also sex-biased (SB?) in an analysis performed by Cauret et al. 2023.
(XLSX)

S2 Table Differentially expressed genes in same sex comparisons between knockout and wildtype tadpole gonads for X. laevis dmrt1. S and dmrt1.L lines using counts from Kallisto and analysis with DeSeq2 (Analysis 2). The four analyses performed include comparisons of female or male dmrt1S knockouts to same-sex wildtypes (dmrt1.S females, dmrt1.S males, respectively) and comparisons of female or male dmrt1.L knockouts to same-sex wildtypes (dmrt1.L females, dmrt1.L males, respectively). Negative log2 fold change (logFC) means expression is higher in the wildtype compared to the knockout; positive values indicate the opposite. Other informatiojn includes the log fold change standard error (lfcSE), Stat is the logFC/lfcSE; the p value (PValue) and Benjamini-Hochberg adjusted p valuie (padj), the chromosome and subgenome for each gene, the gene acronym for Xenopus (Xen_acro) and humans (Hum_acro), and whether each differentially expressed gene was also sex-biased (SB?) in an analysis performed by Cauret et al. 2023.
(XLSX)

S3 Table Analysis 1 gene ontology analysis of differentially expression genes in the developing gonads for three knockout lines (dmrt1L females, dmrt1L males, dmrt1S females, dmrt1S males) compared to same-sex wildtype siblings, and for wildtype males compared to wildtype females (MF1, MF2, MF3). Results are listed for three gene ontology categories (biological process, molecular function, cellular component); subcategories with significant enrichment follow their parent category and are indicated with “>”s, which reflect the degree of nestedness. For each gene and analyis, the number of differentially expressed genes is indicated (# DE) and NS indicates no significant enrichment. Analyses were performed for one quantification method (STAR) and one method for analysis of differential expression (edgeR) and the false detection rate P-value is indicated for each significantly enriched annotation (FDR). Because a putative human ortholog was not identified for some transcripts (Table S1), the number of genes used in the gene ontology analysis was generally lower than the number of differentially expressed genes.
(XLSX)

S4 Table Analysis 2 gene ontology analysis of differentially expression genes in the developing gonads for three knockout lines (dmrt1L females, dmrt1L males, dmrt1S females, dmrt1S males) compared to same-sex wildtype siblings, and for wildtype males compared to wildtype females (MF1, MF2, MF3). Results are listed for three gene ontology categories (biological process, molecular function, cellular component); subcategories with significant enrichment follow their parent category and are indicated with “>”s, which reflect the degree of nestedness. For each gene and analyis, the number of differentially expressed genes is indicated (# DE) and NS indicates no significant enrichment. Analyses were performed for one quantification method (Kallisto) and one method for analysis of differential expression (DeSeq2) and the false detection rate P-value is indicated for each significantly enriched annotation (FDR). Because a putative human ortholog was not identified for some transcripts (Table S1), the number of genes used in the gene ontology analysis was generally lower than the number of differentially expressed genes.
(XLSX)

S5 Table Permutations of sex-related genes in X. laevis fail to recover significant evidence for masculinization or feminization of knockout transcriptomes in the developing mesonephros/gonad for females or males, respectively. Four same-sex analyses of differential expression for dmrt1.L and dmrt1.S (dmrt1L_FF, dmrt1L_MM, dmrt1S_FF, dmrt1S_MM) were compared to three independent comparisons of wildtype male and female transcriptomes (MF1, MF2, MF3).
(XLSX)

S6 Table Guides and primers used in this study; all listed in 5’ to 3’ orientation. Guide target sequences have the PAM site separaated by a dash; a forward slash separates the sequences of each strand of the target. In the guilde oligos, the SP6 promoter sequences are separated from the guide sequence by dashes.
(XLSX)

S7 Table Information on frogs used in this study including individual identification number (ID), Species, knockout line (Line), genotype (wildtype:WT, heterozygous: Het, or homozygous null: KO), sex, data collected (Data), and other information (Notes). Wildtype individuals are siblings of mutant individuals in one of the three lines (dmrt1.L, dmrt1.S, or dmrt1).
(XLSX)

S8 Table Information about 90 sex-related genes considered in this study including the gene acronym (Acronym), name (Name), the gene name in Xenbase (Xenbase), and the nature of sex-relatedness (Category). (XLSX)
