# Supplementary material for: Sex-specific functional evolution of Dmrt1 in African clawed frogs (Xenopus), and the importance of genetic tipping points in developmental biology
Source: PLoS Genet. 2026 Jan 2;22(1):e1011992. doi: 10.1371/journal.pgen.1011992 (PMC12758760; doi:10.1371/journal.pgen.1011992)
Supplement: S1 Text — Fig A. (A) Sanger sequences of wildtype (wt) and homozygous knockout (ko) individual of X. tropicalis (top) and X. laevis (middle, bottom) illustrate loss of function frameshift mutations including a 1 bp deletion in X. tropicalis, and two independent 7 bp deletions in X. laevis dmrt1.L and dmrt1.S. Each mutation is in the coding region and very near the start codon (by interrupting the 26th, 10th, or 11th amino acid out of 337 or 336 in total; see main text). (B) Distributions of exons (gray boxes), introns and flanking non-transcribed regions (black lines), the DM domain (black boxes in exons), and locations of frameshift mutations (red x followed by the first amino acid position that is affected by the mutation). Starts of transcription are indicated with black arrows, including both isoforms of dmrt1.L; start and stop of translation are indicated with a green arrow and “STOP” respectively. The number below exon indicate the number of amino acids encoded in wildtypes. Fig B. Empty oviducts (O) associated with the dissected of the kidney (K) and fat bodies (F) an X. laevis dmrt1.L homozygous knockout female. The ventral surface of the kidney is shown; anterior is on the top of the image. Fig C. Additional examples of testis morphology with labeling and black scale bar following Fig 2, including a wildtype individual (top: individual 185E) and a dmrt1.L homozygous knockout (bottom: individual 1880). A dotted yellow line in each image demarcates a seminiferous tubule. Scale bar is 50μm. Fig D. Scanning electron microscopy images of wildtype sperm (top left) and sperm from X. laevis homozygous knockout for dmrt1.L. Scale bars are 5μm. (DOCX) [file pgen.1011992.s001.docx]

***S1 Text***

***Functional roles of Dmrt1 during development***

***Germ cell specification, commitment, migration, meiotic entry***

During mammalian fetal development, the indifferent gonad consists of the coelomic epithelium, mesenchymal cells, and germ cells, and forms on the ventromedial surface of the embryonic kidney (the mesonephros) [reviewed in 1]. Germ cells are cells that give rise to gametes (egg, sperm) and are distinguished from other tissues by their capacity to undergo meiosis. Germ cells must initially divide and differentiate mitotically and migrate during development into the genital ridge (the somatic precursor of the gonads) before the germ cells undergo a turning point called “meiotic entry” where their cell division can then occur by meiosis to generate gametes. Germ cells originate from primordial germ cells, whose differentiation from somatic cells is one of the earliest cell fate commitments in animal development. Germ cell commitment occurs via at least two distinct mechanisms: epigenesis and preformation [2, 3]. In mammals (but not fish and anurans), global demethylation occurs after fertilization, thereby resetting epigenetic marks [4]. In human cell lines, epigenetic resetting of migrating primordial germ cells is promoted by *dmrt1* in combination with other factors via induction of 5-hydromethylcytosine, and germline commitment is also facilitated by *dmrt1* via induction of methylation-sensitive genes such as *Dazl* [5].

In many animals, there are striking differences between females and males in the developmental timing of meiotic entry [2, 6]. In human females, for example, meiotic entry occurs in utero and is then arrested in prophase I until ovulation happens (which for an individual egg could be anytime between puberty and menopause). In human males, meiotic entry occurs long after birth at puberty and meiosis continues throughout life [7]. In birds, meiotic entry also occurs embryonically in females but later in males [8]. The developmental timing of meiosis entry is precipitated by multiple factors, notably including all-*trans* retinoic acid in both sexes [9], and with sex-differences in the developmental timing of exposure to this molecule [6, 10]. In male mice, *Dmrt1* blocks retinoic acid signalling from activating genes that are involved with gonadal feminization [11].

During fetal development of male mice, *dmrt1* represses expression of *Stra8*, which is an inducer of meiosis; conditional deletion of *dmrt1* in undifferentiated spermatogonia prevents production of sperm [12]. In the fetal mouse ovary, *dmrt1* activates *Stra8* [13]; female mice with a homozygous null *dmrt1* mutation are fertile but have fewer ovarian follicles compared to wildtype individuals [13, 14]. These sex-specific consequences of *dmrt1* expression are correlated with sex-differences in the timing of meiotic entry. In humans, sex-biased expression of *dmrt1* is also consistent with a key role in the sex-differences in meiotic entry [15].

***Differentiation and maintenance of germ cells and somatic gonadal tissue***

In mammals, the development of the genital ridge, which is the developmental precursor to the gonads, is governed by steroidogenic factor (*sf1*) and Wilms tumor protein 1 (*wt1*) and loss of function of these genes (and of genes that regulate them such as GATA Binding Protein 4; *gata4*) results in urogenital anomalies, including the adrenal and gonadal agenesis [16-18].

Later in development in mice, estrogen receptors and *Foxl2* help maintain female differentiation of granulosa cells [19, 20]. Differentiation of precursor cells into granulosa cells is initially achieved by *Rspo1* and *Wnt4* ligand signalling through the *β-catenin* transcription factor (*CTNNB1*), but expression of these genes becomes female-biased soon thereafter [21]. Granulosa cells require expression of *Foxl2* to remain differentiated, and deletion of this gene causes adult female to male transdifferentiation [20]. Loss of *dmrt1* causes male to female transdifferentiation and is associated with increased expression of *Foxl2* [22].

In mice, androgen signalling from Sertoli cells is required for generation of mature spermatids, even though germ cells do not express androgen receptors [23]. Sertoli cells also secrete anti-Mullerian hormone which (in mammals) causes the Mullerian duct to degenerate [24]. Sertoli cells also provide an immune function for developing sperm, which is necessary because sperm arise after the immune system is established and are thus not recognized as “self” [25]. Nutrients such as iron are needed due to high mitotic activity and biogenesis of mitochondria in sperm.

*Dmrt1* is also involved with differentiation and maintenance of germ cells and somatic tissues in the gonads. In male mice, *dmrt1* is expressed in the genital ridge and primordial germ cells of both sexes [26, 27], and (postnatally) in Sertoli cells, and spermatogonia [27, 28]. Knockout of *dmrt1* results in improper differentiation of Sertoli and germ cells (Herpin & Schartl, 2011; Kim et al., 2007). In mammals, *Dmrt1* also plays a key role in maintenance of gonadal identity: conditional knock-in of *dmrt1* in adult ovaries leads to development of male Sertoli-like cells, illustrating striking plasticity of gonadal differentiation [29]. In tilapia fish, *dmrt1* is required for testis development [30].

The hypothalamus, pituitary, and gonads are endocrine glands that typically secrete hormones in concert, that then influence further production of hormones in each gland, and help orchestrate developmental processes related to sexual differentiation and reproduction, aging, and immunity. Activation of the hypothalamic-pituitary-gonadal axis occurs three times during human development: initially in utero and coupled with sexual differentiation, and then in the first postnatal months of life (“mini-puberty”) and again with the onset of true puberty [31, 32]. Unsurprisingly, expression of *dmrt1* varies through developmental time, tissues, and between the sexes. In humans, *dmrt1* is expressed early during in utero development in germ cell precursors (oogonia and gonocytes), but only in males after birth, with expression shifting from mostly in the Sertoli cells of the testes during fetal development and mini-puberty to mostly in spermatogonia (undifferentiated male germ cells) after puberty [15]. Deletion of a region of human chromosome 9p that includes *dmrt1* and causes hemizygosity in *dmrt1* is associated with 46,XY feminization and gonadal dysgenesis [33, 34]. A heterozygous nonsynonymous point mutation in the C-terminal DNA recognition helix of *dmrt1* a 46,XY human resulted in a fully feminized external phenotype coupled with complete gonadal dysgenesis [34]. Together, these findings suggest *dmrt1* is haploinsufficient in humans for testicular development [34].

In birds, sex-differences in allelic dosage of *dmrt1* (females have one allele, males have two) governs primary sexual differentiation and *dmrt1* is essential for testis development [35]. However, ovarian development is dependent on synthesis of estrogen and does not require *dmrt1* [35]. Interestingly, secondary sexual differentiation in birds is cell autonomous and not substantially affected by gonadal development [35, 36].

In fruit flies, sex-specific splicing of a *dmrt1* homolog (*doublesex, dsx*) triggers sexual differentiation based on the presence or absence of expression of a protein called transformer, which also undergoes sex specific splicing [37]. In *Caenorhabditis* nematodes, the *dmrt1* homolog (*male abnormal* *3*, *mab-3*) plays an important role in male differentiation through the prevention of yolk production and regulation of male reproductive structures [38].

Sex-specific supporting cells – granulosa cells in females, Sertoli cells in males – play key roles in providing germ cells with nutrients and promoting their differentiation. These cells are developmentally homologous [21, 39] and at least partially derived from coelomic epithelium precursors cells in mice [40] but in chicken are derived from a different tissue type – the gonadal mesenchyme [41]. In the ovary, pre-granulosa cells secrete all-*trans* retinoic acid, which is responsible for meiotic entry of germ cells [42]. Postpubescent Sertoli cells have alternate functional states [mode A and mode B; 43] and those in mode A also express retinoic acid, which promotes differentiation of spermatogonia and is coupled with increased expression of Bone morphogenetic protein 4 (*Bmp4*), glial cell line-derived neurotrophic factor (*GDNF*), and Stimulated by Retinoic Acid gene 8 (*Stra8*) in spermatogonia stem cells [25].

***Hormone secreting cells: theca, Leydig***

In mice, theca cells in the ovary are derived from two populations of precursor cells – one that arises from within the developing gonad and expresses the Wilms tumour 1 (*wt1*) transcription factor, and another mesenchymal population that migrates to the gonad from the mesonephros [44]. Theca cells produce androgens which are converted to estrogens in granulosa cells, which express aromatase (*Cyp19A1*) [45]. Leydig cells secrete activin A, which (in mice) triggers a second wave of proliferation of Sertoli cells (the first wave of proliferation is initiated by a positive feedback loop between Sertoli cells mediated by *Sox9*) [46].

***Genetic triggers for sexual differentiation***

In both sexes, adult gonads carry out crucial roles for generation of gametes and sex hormones [46]. The development and maintenance of the bipotential gonad (the “genital ridge”) appears to be a necessary prelude to sexual differentiation [47]. Once the bipotential gonad has developed, sexual differentiation can be triggered by environmental or genetic queues, and these triggers evolve rapidly in some groups such as vertebrates [48, 49].

With the exception of 17β-hydroxysteroid dehydrogenase 1 (*hsd17b1*) [50], steroid receptors and associated enzymes such as aromatase are typically not known to trigger sexual differentiation, even though they play key roles in sexual differentiation later in development [51]. Similarly, *Sox9* and *Foxl2* are important components of sexual differentiation but have yet to be identified as triggers for sex determination [52]. Sometimes genes whose ancestral function is not sex-related are recruited to become triggers for sex determination. Examples of gene recruitment include *sdY*, which regulates sexual differentiation in salmonid fish, and is a paralog of an immune-related gene called interferon-regulatory factor 9 [53]. Another example of gene recruitment is growth differentiation factor 6 Y (*gdf6Y*), which is the male-determining gene in killifish; *gdf6Y* is a member of the TGF-β signaling pathway but this gene has not otherwise been implicated with gonadal development [54]. Loss of an ancestral sex-related gene (*Sry*) occurred independently at least twice in therian mammals [55-57].

**References**

1. Tanaka SS, Nishinakamura R. Regulation of male sex determination: genital ridge formation and Sry activation in mice. Cellular and Molecular Life Sciences. 2014;71(24):4781–802.

2. Hansen CL, Pelegri F. Primordial Germ Cell Specification in Vertebrate Embryos: Phylogenetic Distribution and Conserved Molecular Features of Preformation and Induction. Frontiers in Cell and Developmental Biology. 2021;9. doi: 10.3389/fcell.2021.730332. PubMed PMID: WOS:000702015900001.

3. Extavour CG, Akam M. Mechanisms of germ cell specification across the metazoans: epigenesis and preformation. Development. 2003;130(24):5869-84. doi: 10.1242/dev.00804. PubMed PMID: WOS:000187400100002.

4. Paranjpe SS, Veenstra GJC. Establishing pluripotency in early development. Biochimica Et Biophysica Acta-Gene Regulatory Mechanisms. 2015;1849(6):626-36. doi: 10.1016/j.bbagrm.2015.03.006. PubMed PMID: WOS:000355895300005.

5. Irie N, Lee SM, Lorenzi V, Xu HQ, Chen JF, Inoue M, Kobayashi T, Sancho-Serra C, Drousioti E, Dietmann S, Vento-Tormo R, Song CX, Surani MA. DMRT1 regulates human germline commitment. Nature Cell Biology. 2023;25(10):1439-+. doi: 10.1038/s41556-023-01224-7. PubMed PMID: WOS:001066016000001.

6. Sou IF, Pryce RM, Tee WW, McClurg UL. Meiosis initiation: a story of two sexes in all creatures great and small. Biochemical Journal. 2021;478(20):3791-805. doi: 10.1042/BCJ20210412. PubMed PMID: WOS:000726836100006.

7. McLaren A. Somatic and germ-cell sex in mammals. Philosophical Transactions of the Royal Society B-Biological Sciences. 1988;322(1208):3-8. doi: 10.1098/rstb.1988.0109. PubMed PMID: WOS:A1988R317300001.

8. Smith CA, Roeszler KN, Bowles J, Koopman P, Sinclair AH. Onset of meiosis in the chicken embryo; evidence of a role for retinoic acid. Bmc Developmental Biology. 2008;8. doi: 10.1186/1471-213X-8-85. PubMed PMID: WOS:000260126200001.

9. Bowles J, Knight D, Smith C, Wilhelm D, Richman J, Mamiya S, Yashiro K, Chawengsaksophak K, Wilson MJ, Rossant J, Hamada H, Koopman P. Retinoid signaling determines germ cell fate in mice. Science. 2006;312(5773):596-600. doi: 10.1126/science.1125691. PubMed PMID: WOS:000237296700050.

10. Teletin M, Vernet N, Yu JS, Klopfenstein M, Jones JW, Féret B, Kane MA, Ghyselinck NB, Mark M. Two functionally redundant sources of retinoic acid secure spermatogonia differentiation in the seminiferous epithelium. Development. 2019;146(1). doi: 10.1242/dev.170225. PubMed PMID: WOS:000455850900007.

11. Minkina A, Matson CK, Lindeman RE, Ghyselinck NB, Bardwell VJ, Zarkower D. DMRT1 Protects Male Gonadal Cells from Retinoid-Dependent Sexual Transdifferentiation. Developmental Cell. 2014;29(5):511-20. doi: 10.1016/j.devcel.2014.04.017. PubMed PMID: WOS:000337644700005.

12. Matson CK, Murphy MW, Griswold MD, Yoshida S, Bardwell VJ, Zarkower D. The Mammalian Doublesex Homolog DMRT1 Is a Transcriptional Gatekeeper that Controls the Mitosis versus Meiosis Decision in Male Germ Cells. Developmental Cell. 2010;19(4):612-24. doi: 10.1016/j.devcel.2010.09.010. PubMed PMID: WOS:000283522700013.

13. Krentz AD, Murphy MW, Sarver AL, Griswold MD, Bardwell VJ, Zarkower D. DMRT1 promotes oogenesis by transcriptional activation of Stra8 in the mammalian fetal ovary. Developmental Biology. 2011;356(1):63-70. doi: 10.1016/j.ydbio.2011.05.658. PubMed PMID: WOS:000292784400006.

14. Raymond CS, Murphy MW, O'Sullivan MG, Bardwell VJ, Zarkower D. DMRT1, a gene related to worm and fly sexual regulators, is required for mammalian testis differentiation. Genes and Development. 2000;14:2587–95.

15. Jorgensen A, Nielsen JE, Jensen MB, Græm N, Rajpert-De Meyts E. Analysis of meiosis regulators in human gonads: a sexually dimorphic spatio-temporal expression pattern suggests involvement of DMRT1 in meiotic entry. Molecular Human Reproduction. 2012;18(11):523-34. doi: 10.1093/molehr/gas030. PubMed PMID: WOS:000310374300002.

16. Kreidberg JA, Sariola H, Loring JM, Maeda M, Pelletier J, Housman D, Jaenisch R. WT-1 is required for early kidney development. Cell. 1993;74(4):679-91. doi: 10.1016/0092-8674(93)90515-R. PubMed PMID: WOS:A1993LU59200011.

17. Hu YC, Okumura LM, Page DC. Gata4 is required for formation of the genital ridge in mice. Plos Genetics. 2013;9(7). doi: 10.1371/journal.pgen.1003629. PubMed PMID: WOS:000322321100031.

18. Zhao LP, Bakke M, Krimkevich Y, Cushman LJ, Parlow AF, Camper SA, Parker KL. Steroidogenic factor 1 (SF1) is essential for pituitary gonadotrope function. Development. 2001;128(2):147-54. PubMed PMID: WOS:000166764100001.

19. Couse JF, Hewitt SC, Bunch DO, Sar M, Walker VR, Davis BJ, Korach KS. Postnatal sex reversal of the ovaries in mice lacking estrogen receptors α and β. Science. 1999;286(5448):2328-31. doi: 10.1126/science.286.5448.2328. PubMed PMID: WOS:000084318500057.

20. Uhlenhaut NH, Jakob S, Anlag K, Eisenberger T, Sekido R, Kress J, Treier AC, Klugmann C, Klasen C, Holter NI, Riethmacher D, Schütz G, Cooney AJ, Lovell-Badge R, Treier M. Somatic Sex Reprogramming of Adult Ovaries to Testes by FOXL2 Ablation. Cell. 2009;139(6):1130-42. doi: 10.1016/j.cell.2009.11.021. PubMed PMID: WOS:000272622800015.

21. Chassot AA, Gillot I, Chaboissier MC. R-spondin1, WNT4, and the CTNNB1 signaling pathway: strict control over ovarian differentiation. Reproduction. 2014;148(6):R97-R110. doi: 10.1530/REP-14-0177. PubMed PMID: WOS:000345914400004.

22. Matson CK, Murphy MW, Sarver AL, Griswold MD, Bardwell VJ, Zarkower D. DMRT1 prevents female reprogramming in the postnatal mammalian testis. Nature (London). 2011;476(7358):101. doi: 10.1038/nature10239. PubMed PMID: BIOSIS:PREV201100543426.

23. De Gendt K, Swinnen JV, Saunders PTK, Schoonjans L, Dewerchin M, Devos A, Tan K, Atanassova N, Claessens F, Lécureuil C, Heyns W, Carmeliet P, Guillou F, Sharpe RM, Verhoeven G. A Sertoli cell-selective knockout of the androgen receptor causes spermatogenic arrest in meiosis. Proceedings of the National Academy of Sciences of the United States of America. 2004;101(5):1327-32. doi: 10.1073/pnas.0308114100. PubMed PMID: WOS:000188796800042.

24. Barrionuevo F, Burgos M, Jiménez R. Origin and function of embryonic Sertoli cells. BioMolecular Concepts. 2011;2011:537-47.

25. Thumfart KM, Mansuy IM. What are Sertoli cells? Historical, methodological, and functional aspects. Andrology. 2023;11(5):849-59. doi: 10.1111/andr.13386. PubMed PMID: WOS:000928816900001.

26. Bellefroid EJ, Leclère L, Saulnier A, Keruzore M, Sirakov M, Vervoort M, De Clercq S. Expanding roles for the evolutionarily conserved <i>Dmrt</i> sex transcriptional regulators during embryogenesis. Cellular and Molecular Life Sciences. 2013;70(20):3829-45. doi: 10.1007/s00018-013-1288-2. PubMed PMID: WOS:000324774000006.

27. Lei N, Hornbaker KI, Rice DA, Karpova T, Agbor VA, Heckert LL. Sex-specific differences in mouse DMRT1 expression are both cell type- and stage-dependent during gonad development. Biology of Reproduction. 2007;77(3):466-75. doi: 10.1095/biolreprod.106.058784. PubMed PMID: WOS:000248953900009.

28. Raymond C, Kettlewell J, Hirsch B, Bardwell VJ, Zarkower D. Expression of Dmrt1 in the genital ridge of mouse and chicken embryos suggests a role in vertebrate sexual development. Developmental Biology. 1999;215:208–20.

29. Lindeman RE, Gearhart MD, Minkina A, Krentz AD, Bardwell VJ, Zarkower D. Sexual Cell-Fate Reprogramming in the Ovary by DMRT1. Current Biology. 2015;25(6):764-71. doi: 10.1016/j.cub.2015.01.034. PubMed PMID: WOS:000351312100027.

30. Qi SS, Dai SF, Zhou X, Wei XY, Chen P, He YY, Kocher TD, Wang DS, Li MH. Dmrt1 is the only male pathway gene tested indispensable for sex determination and functional testis development in tilapia. Plos Genetics. 2024;20(3). doi: 10.1371/journal.pgen.1011210. PubMed PMID: WOS:001194755700002.

31. Lucaccioni L, Trevisani V, Boncompagni A, Marrozzini L, Berardi A, Iughetti L. Minipuberty: Looking Back to Understand Moving Forward. Frontiers in Pediatrics. 2021;8. doi: 10.3389/fped.2020.612235. PubMed PMID: WOS:000613281200001.

32. Kuiri-Hänninen T, Sankilampi U, Dunkel L. Activation of the Hypothalamic-Pituitary-Gonadal Axis in Infancy: Minipuberty. Hormone Research in Paediatrics. 2014;82(2):73-80. doi: 10.1159/000362414. PubMed PMID: WOS:000341584900001.

33. Veitia RA, Nunes M, Quintana-Murci L, Rappaport R, Thibaud E, Jaubert E, Fellous M, McElreavey K, Goncalves J, Silva M, Rodrigues JC, Caspurro M, Boieiro F, Marques R, Lavinha J. Swyer syndrome and 46,XY partial gonadal dysgenesis associated with 9p deletions in the absence of monosomy-9p syndrome. American Journal of Human Genetics. 1998;63(3):901-5. doi: 10.1086/302023. PubMed PMID: WOS:000075919000032.

34. Murphy MW, Lee JK, Rojo S, Gearhart MD, Kurahashi K, Banerjee S, Loeuille GA, Bashamboo A, McElreavey K, Zarkower D, Aihara H, Bardwell VJ. An ancient protein-DNA interaction underlying metazoan sex determination. Nature Structural & Molecular Biology. 2015;22(6):442-U26. doi: 10.1038/nsmb.3032. PubMed PMID: WOS:000355620600005.

35. Ioannidis J, Taylor G, Zhao DB, Liu L, Idoko-Akoh A, Gong DQ, Lovell-Badge R, Guioli S, McGrew MJ, Clinton M. Primary sex determination in birds depends on DMRT1 dosage, but gonadal sex does not determine adult secondary sex characteristics. Proceedings of the National Academy of Sciences of the United States of America. 2021;118(10). doi: 10.1073/pnas.2020909118. PubMed PMID: WOS:000627429100077.

36. Zhao D, McBride D, Nandi S, McQueen HA, McGrew MJ, Hocking PM, Lewis PD, Sang HM, Clinton M. Somatic sex identity is cell autonomous in the chicken. Nature. 2010;464(7286):237-42.

37. Rideout EJ, Dornan AJ, Neville MC, Eadie S, Goodwin SF. Control of sexual differentiation and behavior by the doublesex gene in Drosophila melanogaster  Nature neuroscience. 2010;13(4):458–66.

38. Shen MM, Hodgkin J. mab-3, a gene required for sex-specific yolk protein expression and a male-specific lineage in C. elegans. Cell. 1988;54(7):1019–31.

39. Albrecht KH, Eicher EM. Evidence that Sry is expressed in pre-Sertoli cells and Sertoli and granulosa cells have a common precursor. Developmental Biology. 2001;240(1):92-107. doi: 10.1006/dbio.2001.0438. PubMed PMID: WOS:000172723300007.

40. Rotgers E, Jorgensen A, Yao HHC. At the Crossroads of Fate-Somatic Cell Lineage Specification in the Fetal Gonad. Endocrine Reviews. 2018;39(5):739-59. doi: 10.1210/er.2018-00010. PubMed PMID: WOS:000448057900009.

41. Estermann MA, Williams S, Hirst CE, Roly ZY, Serralbo O, Adhikari D, Powell D, Major AT, Smith CA. Insights into Gonadal Sex Differentiation Provided by Single-Cell Transcriptomics in the Chicken Embryo. Cell Reports. 2020;31(1). doi: 10.1016/j.celrep.2020.03.055. PubMed PMID: WOS:000524976500012.

42. Baumgarten SC, Stocco C. Granulosa Cells. In: Crum CP, Lee KR, Nucci MR, Granter SR, Howitt BE, Parast MM, et al., editors. Diagnostic gynecologic and obstetric pathology E-book Elsevier Health Sciences, 2017. E-book: Elsevier Health Sciences; 2018.

43. Linder CC, Heckert LL, Roberts KP, Kim KH, Griswold MD, Robaire B. Expression of receptors during the cycle of the seminiferous epithelium. Male Germ Cell : Spermatogonium To Fertilization. 1991;637:313-21. doi: 10.1111/j.1749-6632.1991.tb27318.x. PubMed PMID: WOS:A1991BW41F00022.

44. Liu C, Peng J, Matzuk MM, Yao HHC. Lineage specification of ovarian theca cells requires multicellular interactions via oocyte and granulosa cells. Nature Communications. 2015;6. doi: 10.1038/ncomms7934. PubMed PMID: WOS:000353704400003.

45. Edson MA, Nagaraja AK, Matzuk MM. The Mammalian Ovary from Genesis to Revelation. Endocrine Reviews. 2009;30(6):624-712. doi: 10.1210/er.2009-0012. PubMed PMID: WOS:000270708100003.

46. Ungewitter EK, Yao HHC. How to Make a Gonad: Cellular Mechanisms Governing Formation of the Testes and Ovaries. Sexual Development. 2013;7(1-3):7-20. doi: 10.1159/000338612. PubMed PMID: WOS:000312010000002.

47. Adolfi MC, Herpin A, Schartl M. The replaceable master of sex determination: bottom-up hypothesis revisited. Philosophical Transactions of the Royal Society B. 2021;376(1832):20200090.

48. Pennell MW, Mank JE, Peichel CL. Transitions in sex determination and sex chromosomes across vertebrate species. Molecular Ecology. 2018;27(19):3950–63.

49. Stoeck M, Kratochvil L, Kuhl H, Rovatsos M, Evans BJ, Suh A, Valenzuela N, Veyrunes F, Zhou Q, Gamble T, Capel B, Schartl M, Guiguen Y. A brief review of vertebrate sex evolution with a pledge for integrative research: towards 'sexomics'. Philosophical Transactions of the Royal Society of London B Biological Sciences. 2021;376(1832):Article No.: 20200426.

50. Koyama T, Nakamoto M, Morishima K, Yamashita R, Yamashita T, Sasaki K, Kuruma Y, Mizuno N, Suzuki M, Okada Y, Ieda R, Uchino T, Tasumi S, Hosoya S, Uno S, Koyama J, Toyoda A, Kikuchi K, Sakamoto T. A SNP in a Steroidogenic Enzyme Is Associated with Phenotypic Sex in Seriola Fishes. Current Biology. 2019;29(11):1901-+. doi: 10.1016/j.cub.2019.04.069. PubMed PMID: WOS:000470902000051.

51. Curzon AY, Shirak A, Ron M, Seroussi E. Master-Key Regulators of Sex Determination in Fish and Other Vertebrates-A Review. International Journal of Molecular Sciences. 2023;24(3). doi: 10.3390/ijms24032468. PubMed PMID: WOS:000930791100001.

52. Pan QW, Kay T, Depincé A, Adolfi M, Schartl M, Guiguen Y, Herpin A. Evolution of master sex determiners: TGF-β signalling pathways at regulatory crossroads. Philosophical Transactions of the Royal Society B-Biological Sciences. 2021;376(1832):20200091.

53. Yano A, Guyomard R, Nicol B, Jouanno E, Quillet E, Klopp C, Cabau C, Bouchez O, Fostier A, Guiguen Y. An immune-related gene evolved into the master sex-determining gene in rainbow trout, Oncorhynchus mykiss Current Biology. 2012;22(15):1423–8.

54. Reichwald K, Petzold A, Koch P, Downie BR, Hartmann N, Pietsch S, Baumgart M, Chalopin D, Felder M, Bens M, Sahm A, Szafranski K, Taudien S, Groth M, Arisi I, Weise A, Bhatt SS, Sharma V, Kraus JM, Schmid F, Priebe S, Liehr T, Görlach M, Than ME, Hiller M, Kestler HA, Volff JN, Schartl M, Cellerino A, Englert C, Platzer M. Insights into Sex Chromosome Evolution and Aging from the Genome of a Short-Lived Fish. Cell. 2015;163(6):1527-38. doi: 10.1016/j.cell.2015.10.071. PubMed PMID: WOS:000366044800023.

55. Just W, Rau W, Vogel W, Akhverdian M, Fredga K, Graves JAM, Lyapunova E. Absence of Sry in species of the vole Ellobius. Nature. 1995;11:117–8.

56. Kuroiwa A, Ishiguchi Y, Yamada F, Shintaro A, Matsuda Y. The process of a Y-loss event in an XO/XO mammal, the Ryukyu spiny rat. Chromosoma. 2010;119:519–26.

57. Mulugeta E, Wassenaar E, Sleddens-Linkels E, van Ijcken WFJ, Heard E, Grootegoed JA, Just W, Gribnau J, Baarends WM. Genomes of <i>Ellobius</i> species provide insight into the evolutionary dynamics of mammalian sex chromosomes. Genome Research. 2016;26(9):1202-10. doi: 10.1101/gr.201665.115. PubMed PMID: WOS:000382421000006.

**Supplementary Figures**

Fig A. (A) Sanger sequences of wildtype (wt) and homozygous knockout (ko) individual of *X. tropicalis* (top) and *X. laevis* (middle, bottom) illustrate loss of function frameshift mutations including a 1 bp deletion in *X. tropicalis*, and two independent 7 bp deletions in *X. laevis dmrt1.L* and *dmrt1.S*. Each mutation is in the coding region and very near the start codon (by interrupting the 26^th^, 10^th^, or 11^th^ amino acid out of 337 or 336 in total; see main text). (B) Distributions of exons (gray boxes), introns and flanking non-transcribed regions (black lines), the DM domain (black boxes in exons), and locations of frameshift mutations (red x followed by the first amino acid position that is affected by the mutation). Starts of transcription are indicated with black arrows, including both isoforms of *dmrt1.L*; start and stop of translation are indicated with a green arrow and “STOP” respectively. The number below exon indicate the number of amino acids encoded in wildtypes.

Fig B. Empty oviducts (O) associated with the dissected of the kidney (K) and fat bodies (F) an *X. laevis dmrt1.L* homozygous knockout female. The ventral surface of the kidney is shown; anterior is on the top of the image.

Fig C. Additional examples of testis morphology with labeling and black scale bar following Fig 2, including a wildtype individual (top: individual 185E) and a *dmrt1.L* homozygous knockout (bottom: individual 1880). A dotted yellow line in each image demarcates a seminiferous tubule. Scale bar is 50μm.

Fig D. Scanning electron microscopy images of wildtype sperm (top left) and sperm from *X. laevis* homozygous knockout for *dmrt1.L*. Scale bars are 5μm.
